# Distinguishing cardiac myxomas from cardiac thrombi by a radiomics signature based on cardiovascular contrast-enhanced computed tomography images

**DOI:** 10.1186/s12872-021-01961-3

**Published:** 2021-03-25

**Authors:** Wen-lei Qian, Yu Jiang, Xi Liu, Ying-kun Guo, Yuan Li, Xin Tang, Zhi-gang Yang

**Affiliations:** 1 Department of Radiology, West China Hospital, Sichuan University, 37# Guo Xue Xiang, Chengdu, 610041 Sichuan China; 2 Key Laboratory of Carcinogenesis and Translational Research (Ministry of Education), Department of Radiology, Peking University Cancer Hospital and Institute, No. 52 Fu Cheng Road Hai Dian District, Beijing, 100142 China; 3 Department of Radiology, Key Laboratory of Obstetric and Gynecologic and Pediatric Diseases and Birth Defects of Ministry of Education, West China Second University Hospital, Sichuan University, 20# South Renmin Road, Chengdu, 610041 Sichuan China

**Keywords:** Radiomics signature, Cardiac myxoma, Cardiac thrombi, Differential diagnosis

## Abstract

**Background:**

Cardiac myxomas (CMs) and thrombi are associated with high morbidity and mortality. These two conditions need totally different treatments. However, they are difficult to distinguish using naked eye. In clinical, misdiagnoses occur now and then. This study aimed to compare the characteristics of CMs and cardiac thrombi and investigate the value of a radiomics signature in distinguishing CMs from cardiac thrombi, based on cardiovascular contrast-enhanced computed tomography (CECT) images.

**Methods:**

A total of 109 patients who had CMs (n = 59) and cardiac thrombi (n = 50) were enrolled in this retrospective study from 2009 to 2019. First, the lesion characteristics of cardiovascular CECT images were documented and compared by two radiologists. Then all patients were randomly allotted to either a primary group or a validation group according to a 7:3 ratio. Univariate analysis and the least absolute shrinkage and selection operator were used to select robust features. The best radiomics signature was constructed and validated using multivariate logistic regression. An independent clinical model was created for comparison.

**Results:**

The best radiomics signature was developed using eight selected radiomics. The classification accuracies of the radiomics signature were 90.8% and 90.9%, and the area under the receiver operating characteristic curves were 0.969 and 0.926 in the training and testing cohorts, respectively. Cardiovascular CECT images showed that the two diseases had significant differences in location, surface, Hydrothorax, pericardial effusion and heart enlargement. The naked eye findings were used to create the clinical model. All metrics of the radiomics signature were higher than those of clinical model.

**Conclusions:**

Compared with clinical model, the radiomics signature based on cardiovascular CECT performed better in differentiating CMs and thrombi, suggesting that it could help improving the diagnostic efficiency.

**Supplementary Information:**

The online version contains supplementary material available at 10.1186/s12872-021-01961-3.

## Introduction

Cardiac tumors are rare, and three-quarters of these tumors are benign. Among them, cardiac myxomas (CMs) represent the vast majority of primary benign cardiac tumors [1, 2]. CM is a benign tumor that can lead to a lethal outcome and has a slim possibility of malignant transformation [3]. CM is a real tumor originating from undifferentiated mesenchymal cells [1, 4]. Cardiac thrombi often appear in patients suffering from cardiovascular diseases such as atrial fibrillation (AF), myocardial infarction, and heart failure [5, 6]. A thrombus consists of insoluble protein fibrin, platelets, white cells, and red cells [7]. CMs and cardiac thrombi are totally different in their essence and are treated in entirely different ways. Because CMs can be lethal and have a slim chance of becoming malignant, patients with CMs need surgical therapy, while patients who have cardiac thrombi might only need anticoagulant therapy or cannot bear operative procedures because of the threat of thromboembolism or patients being poor situation.

Thus, it is very important to diagnose these two conditions correctly. However, in some aspects, they resemble each other. The symptoms of CMs are diverse and nonspecific [8]. These two conditions share some same symptoms. Besides, the characteristics of their radiological images are similar. Both of them manifest as a filling defect in the cardiac chambers [9], making it difficult to arrive at the correct diagnosis when only using the naked eye. Meanwhile, biopsies are hard to get before surgery due to the special anatomical location. Thus, in clinical settings, misdiagnoses occur, and with them comes incorrect treatment [10, 11].

Radiomics is a high-throughput extraction approach that helps to transform digital images into mineable data. It is a helpful quantitative way to improve diagnostic and predictive accuracy, providing a powerful tool in modern medicine [12]. Existing work has already demonstrated that artificial intelligence (AI) can be used to diagnose cardiovascular conditions such as coronary artery disease, poor cardiac function, and so on [13]. However, there have been no reports about applying radiomics (a branch of AI) to cardiac masses. In the meantime, work comparing CMs and cardiac thrombi are limited by small sample sizes [14, 15]. Therefore, this study aimed to compare the characteristics of CMs and cardiac thrombi in cardiovascular contrast-enhanced computed tomography (CECT) images and develop a radiomics signature that can distinguish CMs from cardiac thrombi.

## Methods

### Patients

The cardiovascular CECT datasets were consecutively searched from December 2009 to November 2019. The inclusion criteria for the myxoma group were as follow: (1) patients with CM histologically documented after surgery and (2) patients who underwent cardiovascular CECT in our hospital before surgery. The exclusion criteria were as follows: (1) image quality that was inadequate for delineating target region and (2) incomplete patient medical records. The inclusion criteria for the thrombus group were as follows: (1) patients who went through surgical removal of the thrombus, transesophageal echocardiography (TEE), or transthoracic echocardiography (TTE) in our hospital and were diagnosed with cardiac thrombus; (2) patients with a thrombus who had surgery for other indications should have had cardiovascular CECT in our hospital before surgery; (3) patients with a time interval between cardiovascular CECT and TEE or TTE that was less than 1 week due to the thrombus peculiarity of disappearing quickly once anticoagulant therapy starts; and (4) patients with thrombus with a significant size reduction or disappearance after anticoagulant therapy, which was confirmed on imaging. The exclusion criteria were as follows: (1) imaging quality was inadequate for delineating the target region and (2) incomplete patient medical records.

Finally, a total of 109 patients were consecutively recruited for this study. Fifty-nine patients corresponded to 59 CMs (mean age, 61.6 ± 12.6 years, ranging from 16 to 80 years; female, 33). Fifty patients corresponded to 50 cardiac thrombi (mean age, 58.5 ± 14.5, ranging from 15 to 97 years; female, 23).

First, all CMs and thrombi were reviewed three-dimensionally on a workstation (Syngo-Imaging; Siemens Medical Solution Systems, Forchheim, Germany) and compared carefully by two radiologists without knowing any information of patients. All discrepancies between the two observers were settled by consensus.

Second, all 109 lesions were randomly divided into either the training group or the testing group according to a 7:3 ratio. As a result, 76 lesions and the remaining 33 lesions were allocated to the training group and the testing group, respectively. Meanwhile, basic patient information was collected from the admission records.

Third, after meeting the inclusion and exclusion criteria, the remaining cardiovascular CECT images were retrieved from a picture archiving and communication system (Syngo-Imaging) for feature extraction. When patients had multiple CT examination records, the most recent record was selected. Detailed scanning protocol information is shown in Additional file 1.

**Fig. 1:**
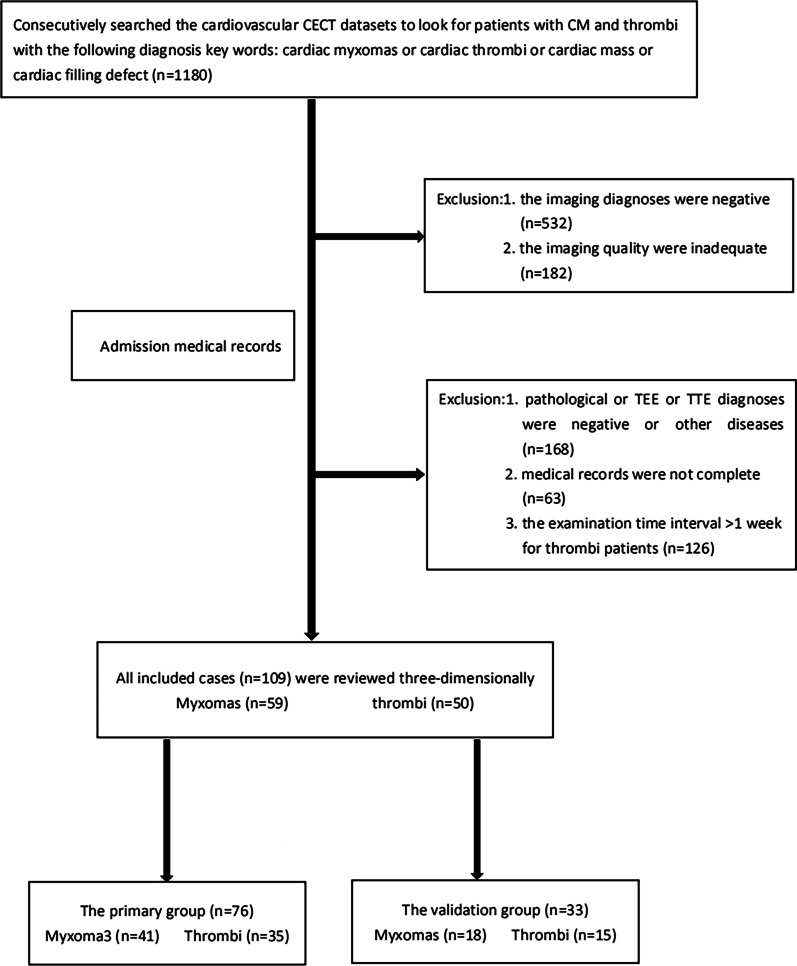
Flow chart of patient recruitment in this study

Finally, referred to a classic radiomics method [16], a radiomics signature and a clinical model were constructed and validated. The specific steps were as follow. The flow chart of this study is shown in Fig. 1.

### Image segmentation and preprocessing

First, all images were loaded into IBEX (β1.0, http://bit.ly/IBEX_MDAnderson], an open infrastructure software platform that streamlines common radiomics workflow tasks. Second, two radiologists with two and five years of experience, respectively, in cardiovascular imaging, independently delineated the region of interests (ROIs) manually without knowing any patient information. Third, a radiologist with 35 years of experience in cardiovascular imaging compared the two ROI groups delineated by the two other radiologists and decided the best group using the next step. Resampling was used as preprocessing steps to ensure repeatability. Resampling was performed to obtain a voxel size of 0.4 × 0.4 × 0.4 mm^3^ via a trilinear interpolation before the feature calculation.

### Feature extraction, dimensionality reduction, and radiomics feature selection

Four commonly used feature groups, shape, intensity histogram, gray-level co-occurrence matrix (GLCM), and gray-level run-length matrix (GLRLM), were extracted from IBEX (Additional file 1: Table 1). A total of 430 radiomics features were extracted from the cardiovascular CECT images. The missing values of all radiomics features were replaced by a value of zero. Z-score was performed to reduce the features’ variable magnitudes by scaling values to a mean of 0 and a standard deviation of 1. To reduce the dimensionality and the bias when building the model, three steps were applied to pick out the robust features in the training group. First, an independent sample t-test or the Mann–Whitney U test was used to select potential useful features. Features were abandoned if they did not meet either of these tests. Second, the least absolute shrinkage and selection operator (LASSO) with five-fold cross-validation was adopted to select features and reduce the dimensionality. The prediction accuracy and interpretability of the model were enhanced by performing variable selection and regularization. The minimum criteria were used to tune the regularization parameter (λ) and for feature selection. Finally, the selected features were tested by Spearman correlation coefficients to avert the latent severe linear dependence. If features were deemed to have severe linear dependence (the correlation coefficients were 0.90–1.00), one of the two features would be chosen to use finally.

### Development of the optimal radiomics signature

**Fig. 2:**
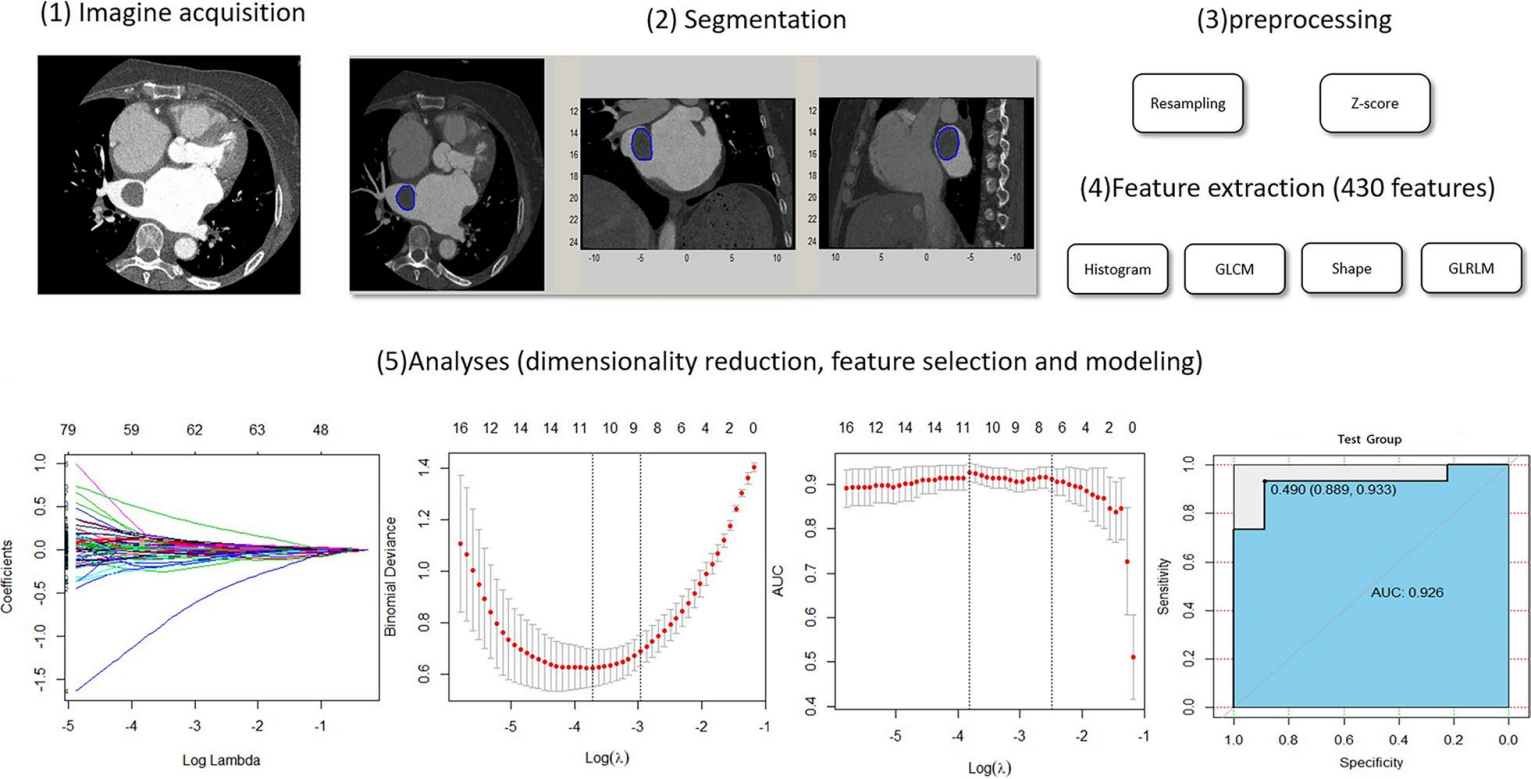
Framework of this study

Logistic regression, a classic machine learning (ML) method, and multivariable binary logistic regression with backward stepwise selection were used to construct a linear classifier. The area under the receiver operating characteristic curves (AUCs) was a main index to assess the optimal radiomics signature. Other discrimination indicators were also calculated, including positive predictive value (PPV), negative predictive value (NPV), specificity, sensitivity and predictive accuracy. After the radiomics signature was constructed in the training group, all the testing group data were put into the model to validate the diagnostic efficiency and accuracy of the model (Fig. 2]. Then an independent clinical model was developed using the same way as above with all naked eye findings for comparison the values of the two models.

### Statistics

During the construction of the radiomics signature, the R language software (Version 3.6.1, https://www.r-project.org/] was used for all statistical analyses. The Shapiro–Wilk test was used to test whether the variables were normal distributions. Bartlett’s test was used to assess the homogeneity of variance. The “lme4” and “psych” packages were used for the intra-class correlation coefficient (ICC). The “glmnet” and “pROC” packages were used for LASSO regression. Basic clinical data were analyzed by Statistical Package for the Social Sciences software (version 25.0). Clinical characteristics were measured based on the variable type. Categorical variables were measured as percentages, and Fisher’s exact or chi-square test was used for comparison, depending on the expected frequencies. Continuous variables were recorded as mean values or medians, and were compared by independent t-tests (normally distributed continuous variables) or the Mann–Whitney U tests (non-normally distributed continuous variables). A two-tailed *p* value < 0.05 was considered statistically significant in all statistical analyses.

## Results

### Clinical characteristics

Patients’ characteristics in the training group and the validation group are presented in Table 1. There was no significant difference between the training group and validation group in terms of sex, age, or clinical manifestations. Among the naked eye findings, calcification, location, and density uniformity had no significant difference either. While heart enlargement was significantly different in both groups (*p* < 0.05), hydrothorax (*p* = 0.030) and pericardial effusion (*p* = 0.020) were significantly different in the validation group, but had no significant difference in the training group. Patients’ medical records were significantly different in two cohorts (*p* = 0.011, *p* = 0.010, respectively).

**Table 1:** Characteristics of patients in the training and validation cohorts

	The primary group		*p*	The validation group		*p*
	Myxoma (41)	Thrombus (35)		Myxoma (18)	Thrombus (15)	
Sex			0.684			0.373
Female	23 (56.1)	17 (48.6)		10 (55.6)	6 (40.0)	
Male	18 (43.9)	18 (51.4)		8 (44.4)	9 (60.0)	
Age(years)	60.98 ± 12.62	56.38 ± 15.35	0.054	62.94 ± 12.92	62.27 ± 12.20	0.873
Clinical manifestation			0.146			0.061
Cardiac signs	27 (55.1)	30 (73.2)		9 (45.0)	13 (86.7)	
Embolism	12 (24.5)	9 (22.0)		6 (30.0)	2 (13.3)	
Constitutional symptoms	3 (6.1)	1 (2.4)		2 (10.0)	0 (0.0)	
No symptom	7 (14.3)	1 (2.4)		3 (15.0)	0 (0.0)	
Medical records			0.011^*^			0.010^*^
Diabetes mellitus	3 (6.7)	3 (3.1)		4 (26.7)	4 (10.5)	
Hypertension	9 (20.0)	7 (7.1)		5 (33.3)	5 (13.2)	
Atrial fibrillation	4 (8.9)	15 (15.3)		0 (0.0)	12 (31.6)	
Rheumatic heart disease	2 (4.4)	28 (28.6)		0 (0.0)	5 (13.2)	
Heart valves obstruction	8 (17.8)	14 (14.3)		1 (6.7)	3 (7.9)	
Heart valves insufficiency	12 (26.7)	19 (19.4)		5 (33.3)	5 (13.2)	
Others	7 (15.6)	12 (12.2)		0 (0.0)	4 (10.5)	
Heart rate	82.00 ± 9.26	89.51 ± 21.65	0.076	82.17 ± 12.61	87.40 ± 13.34	0.244
Cardiac murmur	19 (46.3)	14 (40.0)	0.373	4 (26.7)	7 (46.7)	0.133
Naked eye findings						
Calcification	8 (19.5)	4 (11.4)	0.367	2 (11.1)	3 (20.0)	0.639
Hydrothorax	4 (9.8)	9 (25.7)	0.066	1 (5.6)	6 (40.0)	0.030^*^
Pericardial effusion	9 (22.0)	13 (37.1)	0.146	2 (11.1)	8 (53.3)	0.020^*^
Heart enlargement	25 (61.0)	29 (85.3)	0.036^*^	8 (44.4)	12 (80.0)	0.037^*^
Homogeneous density	27 (65.9)	26 (74.3)	0.425	12 (66.7)	11 (73.3)	0.722
Surface			0.041^*^			0.048^*^
Coarse	24 (58.5)	12 (34.3)		11 (61.1)	4 (26.7)	
Smooth	17 (41.5)	23 (65.7)		7 (38.9)	11 (73.3)	
Location			0.498			0.375
LA	37 (90.2)	29 (82.9)		16 (88.9)	11 (73.3)	
RA	4 (9.8)	6 (17.1)		2 (11.1)	4 (26.7)	

^*^p value < 0.05; ages and heart rate are shown as mean ± standard deviation; other data are the number of patients with the percentage in Parentheses

Cardiac signs included: Dyspnea, palpitations, malaise or syncope, chest pain or discomfort, dizziness, lower limb edema and hemoptysis

Constitutional symptoms included: fever, weight loss, anemia, or pseudo-connective tissue disease signs

Others included: Patent foramen ovale, Pulmonary hypertension, cardiomyopathy and arrhythmia. LA: left atrium, RA: right atrium

**Fig. 3:**
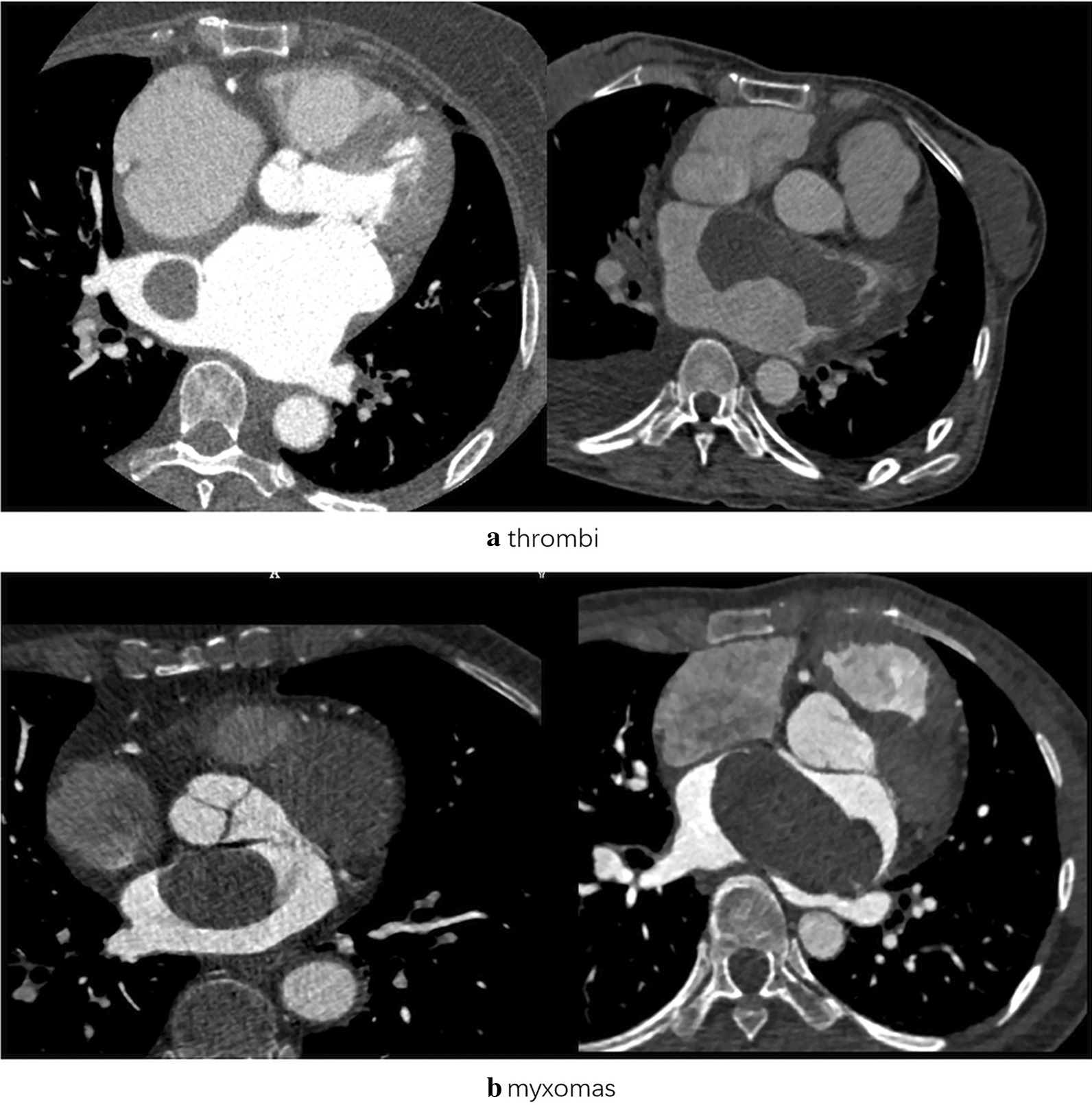
The cardiovascular CECT Images. **a** thrombi, **b** myxomas. Both the two diseases present as filling-defects in the left atrium and mimic each other sometimes

For comparison of CM and thrombus with the naked eye, significant differences were observed in the adjacent structures. The thrombus group had more hydrothorax (30.0% vs. 8.5%, *p* = 0.004), pericardial effusion (42.0% vs. 18.6%, *p* = 0.008), and heart enlargement (82.0% vs. 55.9%, *p* = 0.004). For lesions, lesion surface and location were significantly different between the two groups (*p* = 0.004, *p* = 0.000, respectively). The CM group had more coarse surfaces (59.3%) while the thrombus group had more smooth surfaces (68%). More thrombi (48%) were located in the Left atrial appendage (LAA), while most CMs (88.1%) are in left atrial (LA). Calcification (*p* = 0.672), homogeneous density (*p* = 0.371), and enhanced CT value (0.370) showed no significant difference in the two cohorts (Fig. 3]. More information is shown in Table 2.

**Table 2:** characteristics of CMs and cardiac thrombi based on the cardiovascular CECT

	Myxoma (n = 59)	Thrombus (n = 50)	*p*
Naked eye findings			
Calcification	10 (16.9)	7 (17.1)	0.672
Hydrothorax	5 (8.5)	15 (30.0)	0.004^*^
Pericardial effusion	11 (18.6)	21 (42.0)	0.008^*^
Heart enlargement	33 (55.9)	41 (82.0)	0.004^*^
Homogeneous density	39 (66.1)	37 (74.0)	0.371
Enhanced CT value/HU	54.35 ± 19.10	58.52 ± 22.59	0.370
Surface			0.004^*^
Coarse	35 (59.3)	16 (32.0)	
Smooth	24 (40.7)	34 (68.0)	
Location			0.000^*^
LA	52 (88.1)	17 (34.0)	
LAA	1 (1.7)	24 (48.0)	
RA	6 (10.2)	9 (18.0)	

^*^p value < 0.05; enhanced CT value is shown as mean ± standard deviation; other data are the number of patients with the percentage in parentheses. LA: left atrium, LAA: left atrial appendage, RA: right atrium.

### Inter-observer agreement

The ICC of the 430 radiomics features ranged from 0.03 to 0.99 (mean ICC = 0.98). An ICC score greater than 0.75 was considered a satisfactory agreement. The ICC scores of two features were less than 0.75 and were excluded. Finally, 428 radiomics features were included in the following calculation.

### Dimensionality reduction and feature selection

**Fig. 4:**
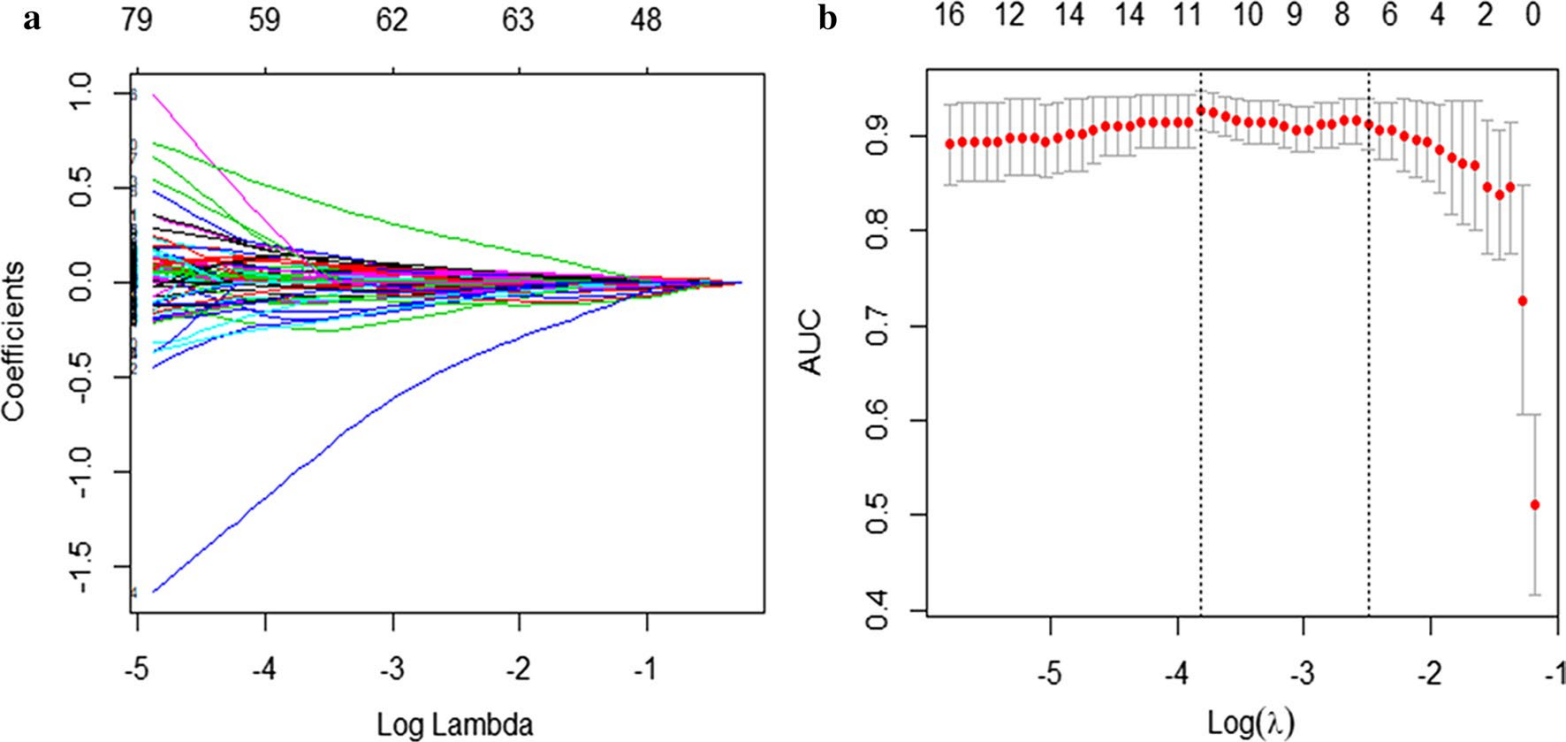
Feature selection using the LASSO regression method. **a** The 5- fold cross validation was used to select the optimal parameters (Lambda, λ = 0.020). **b** The AUC was plotted versus log (Lambda) by using the minimum standard (left line) and the 1 standard error of minimum standard (1 − SE standard, right line) to draw the vertical line with the best value. The minimum standard was used according to the 5-fold cross validation. Eleven features were chosen after LASSO regression

There were 97 radiomics features showing a normal distribution with homogenization, and 85 of them were significantly different based on independent sample t-tests. The remaining features were tested by Mann–Whitney U tests; 236 features were significantly different. Finally, a total of 321 radiomics features were used for LASSO regression, and 11 radiomics features with non-zero coefficients were chosen, with the best-tuned regularization parameter λ of 0.020 under the minimum criteria found by five-fold cross-validation (Fig. 4]. Four pairs of features showed a strong positive correlation when tested by Spearman correlation coefficients (Additional file 1: Table 2). As a result, three features were excluded, and eight features were left (Additional file 1: Table 3).

### Construction of the optimal radiomics signature

**Fig.5:**
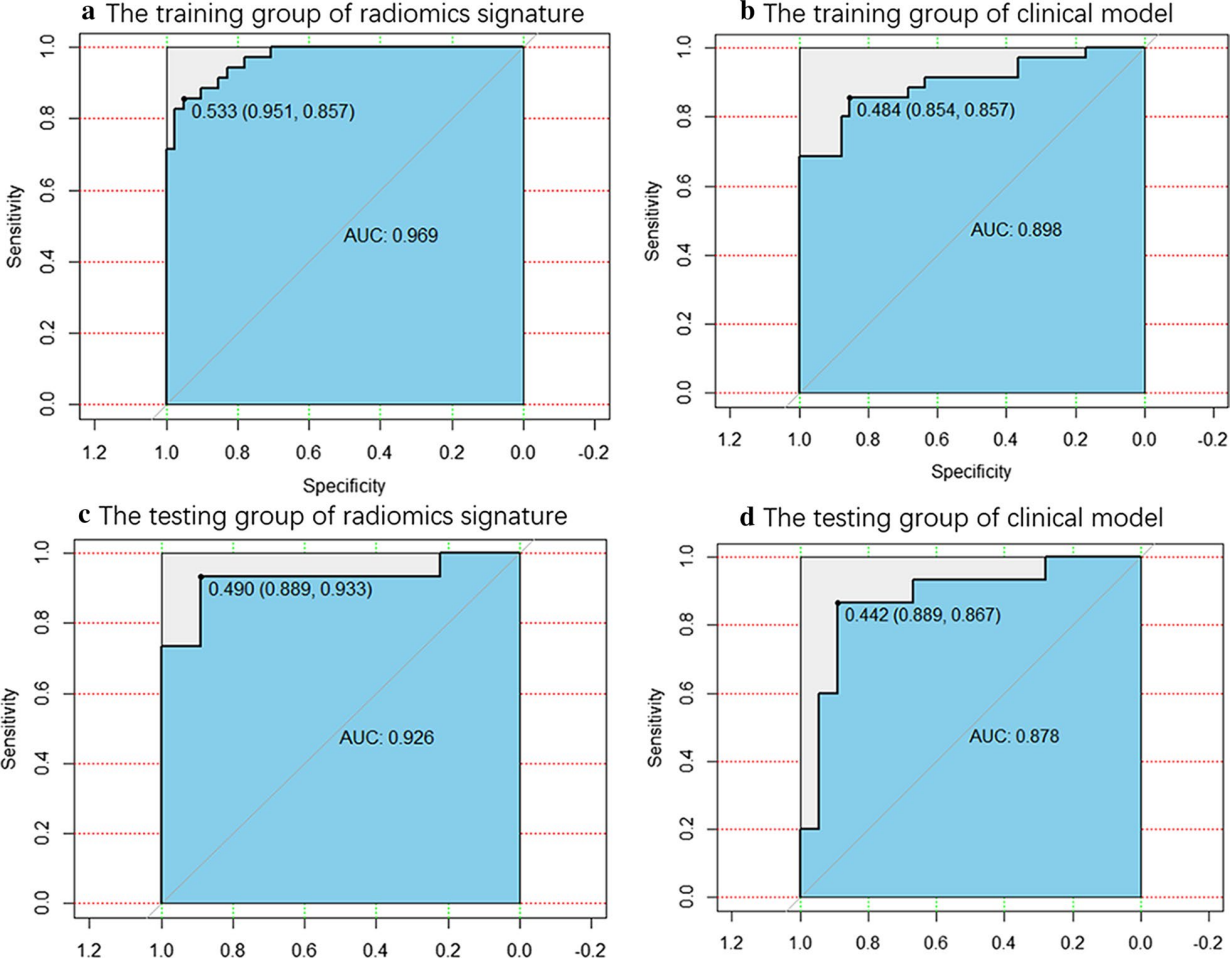
The AUCs and ROCs of two models. **a** The training group of radiomics signature. **b** The training group of clinical model. **c**. The testing group of radiomics signature. **d** The testing group of clinical model. All discrimination metrics of radiomics signature were better than those of clinical model

After Spearman correlation coefficient testing, eight features were selected to construct the predictive radiomics signature. The performance of the radiomics signature was good, with an AUC of 0.969 (95% confidence interval [CI]: 0.939–0.999, Fig. 5a). The classification accuracy, sensitivity, specificity, PPV and NPV were 90.8%, 85.7%, 95.1%, 93.8%, and 88.6%, respectively. Good performance was also observed in the validation group. The AUC was 0.926 (95% CI: 0.819–1.000, Fig. 5c). The accuracy, sensitivity, specificity, PPV, and NPV were 90.9%, 93.3%, 88.9%, 87.5%, and 94.1%, respectively.

For the clinical model, all performance metrics were lower than those of radiomics signature. The AUCs were 0.898 (95% confidence interval [CI]: 0.824–0.973, Fig. 5b) in the training group and 0.878 (95% confidence interval [CI]: 0.749–1.000, Fig. 5d) in the testing group. The classification accuracies were 85.6% and 84.8%, respectively.

## Discussion

Cardiac masses are associated with high morbidity and mortality and imaging plays a vital role in diagnosing and managing them. This study compared CMs and cardiac thrombi (two of the most common cardiac masses) in cardiovascular CECT images with the largest sample size and first explored the value of radiomics in cardiac masses, to our best knowledge. 

In this study, CMs and thrombi were found to have many similarities, which make them hard to be distinguished by only using the naked eyes, though they also have some differences. Many important characteristics of lesions, including calcification, uniformity of density, and enhanced CT value, failed to distinguish CMs from thrombi. The results of the calcification and enhanced CT values were in line with previous studies [14, 15]. The mass surface showed a statistically significant difference with which to differentiate CMs from thrombi. The CM group showed more coarse surfaces, which might be explained by the fact that CM is a real tumor. The growth speeds of tumor cells are various, which leads to a coarse surface. Patients of the thrombi group possessed more hydrothorax, pericardial effusion, and enlarged hearts compared with patients of the CMs group. This might be associated with the fact that patients with a thrombus tend to suffer from AF and rheumatic heart disease, which could lead to thrombi formation. The most common sites of two lesions are different. Many thrombi are located in the LAA. LAA is prone to thrombosis owing to its unique anatomical structure characteristic [17]. Most CMs are located in LA, especially in the atrial septum, which is in line with previous studies [18]. Symptoms were nonspecific in the two cohorts, for all symptoms in the CM group could be observed in the thrombi group. All of these make misdiagnoses are still problems for clinicians. Therefore, it is necessary to find a new method to improve the diagnostic efficiency of radiologists and clinicians.

Thus, we constructed and validated a radiomics signature and a clinical model to distinguish CMs from cardiac thrombi and then compare the diagnostic efficiencies of the two models. The radiomics signature gave a more powerful performance than the clinical model to distinguish CMs and thrombi. All discrimination metrics of the radiomics signature are good and were observed in both the training group and the testing group. Among the eight features selected from radiomics features, cluster prominence, inverse variance and Information Measure Correlation 2 belong to GLCMs, which are second-order statistics. The left five features were first-order statistics that calculated the pixels’ values themselves. As discussed above, the lesion surfaces were significantly different between the two diseases. It was not surprising that three features (compactness, roundness and surface area density), pertained to shape, were selected to construct the discriminative model. Compared to information derived from naked eyes, the combination of first and second order statistics is helpful to analyze the lesion comprehensively.

All discrimination metrics of the clinical model were not as good as the radiomics signature. The clinical model was based on all naked eye findings, including locations, the most important differential diagnostic elements. Visual discoveries were rough and subjective. For example, once a mass was located in the LAA, the verdant radiologists may diagnose it as a thrombus. In fact, it may be CM or other mass. In this study, a myxoma was found to be situated in the LAA. Besides, the clinical model was in the setting that all lesions were identified. While in clinical, the rates of missed diagnosis and misdiagnosis were high. It would further reduce the diagnostic efficiency of radiologists and clinicians. The comparison between the two models showed that radiomics signature was useful for distinguishing CMs and thrombi and more efforts should be made to realize its practicability.

The good performance of the radiomics not only proved the value of radiomics in the heart mass but also indicated that clinicians should take cardiovascular CECT into account when their patients have cardiac masses. The rarity of cardiac tumors, many patients with cardiovascular conditions did not undergo cardiovascular CECT images, and rapid heart movement made it hard to enroll enough images. To the authors’ best knowledge, there was no relevant study about the value of radiomics in cardiac mass. Radiomics has already been applied in other tumors and heart diseases, suggesting that it does have value in diagnosing cardiac masses. For now, CT is the most important source of radiomics. However, when there is a cardiac mass, echocardiography is the first choice for most clinicians for its convenience, inexpensiveness and no radiation [19]. TTE have insurmountable drawbacks such as limited acoustic windows and low tissue resolution, leading to misdiagnosis and missed diagnosis, nevertheless [20]. TEE is a semi-invasive procedure that is a discomfort study and might lead to life-threatening complications [21]. Cardiac MRI has high tissue resolution owing to its multiple sequences. Meanwhile, cardiac MRI could measure cardiac function and mobility of CMs. But MRI is more time and money consuming than CT and could not be used in patients with claustrophobia, metal and contrast contradictions [22, 23]. Cardiovascular CECT has high density resolution and acceptable time and expenditure consumption. With the development of techniques and apparatus, the radiation dose is dramatically reduced and CT applications in the heart diseases has greatly increased [24, 25]. Thus, for the diagnosis and differentiation of the cardiac masses, CT plays an irreplaceable and important role and should be taken into account.

Several limitations of this study should be noted. First, the relatively small sample might influence the radiomics signature. After feature selecting, ML, which needs an enormous amount of data, would be utilized to construct the best classifier. Although our patient number was larger than that in the previous studies, it was relatively small for a radiomics signature. Second, all patients coming from a single-center might influence the robustness of radiomics signature. But this is the first exploration for radiomics value in cardiac masses. A future study with a larger cohort is needed for further validation. Third, due to the nature of retrospective studies, there might be selection bias.

## Conclusion

Compared with clinical model, the radiomics signature based on cardiovascular CECT distinguishes CMs and cardiac thrombi better, indicating a promising future in clinical practice to improve the diagnostic efficiency of radiologists and cardiovascular specialists.

## Supplementary Information

**Additional file 1:** Additional information about scanning protocol and radiomics features.

## Data Availability

The datasets used and/or analyzed during the current study are available from the corresponding author on reasonable request.

## Abbreviations

CECT: Contrast-enhanced computed tomography
CMs: Cardiac myxomas
ROCs: Receiver operating characteristic curves
AUCs: Area Under Curves
ROIs: Region of interests
GLCM: Gray-level co-occurrence matrix
GLRLM: Gray-level run length matrix
LASSO: Least absolute shrinkage and selection operator
PPV: Positive predictive value
NPV: Negative predictive value
ICC: Intraclass correlation coefficient
LA: Left atrium
LAA: Left atrium appendage
