# Supplementary material for: Distinguishing cardiac myxomas from cardiac thrombi by a radiomics signature based on cardiovascular contrast-enhanced computed tomography images
Source: BMC Cardiovasc Disord. 2021 Mar 25;21:152. doi: 10.1186/s12872-021-01961-3 (PMC7993472; doi:10.1186/s12872-021-01961-3)
Supplement: Supplementary file 1 — Additional file 1: Additional information about scanning protocol and radiomics features. [file 12872_2021_1961_MOESM1_ESM.docx]

**Supplementary materials**

**S1: CT image acquisition and reconstruction parameters**

All patients underwent cardiovascular contrast-enhancement computed tomography (CECT) using the following type of scanner: Somatom Definition (Siemens Medical Solutions, Forchheim Germany). Non-ionic iodinated contrast agent (iopamidol, 370 mg of iodine/mL; BraccoSine Pharmaceutical Corp. Ltd, Shanghai, China) was used in all patients with the help of automatic high pressure injector (Medrad Stellant) at a speed of 4.5-5ml/s. The dose was 0.8ml/kg of body weight. After contrast agent injected, a 30ml saline was used at the same rate. Beta blockers was taken in some of the patients as a regular medication before the CT scan, but additional beta blockade was not administered immediately prior to the CT scans. All patients were asked to breath-holding when examination was going on and retrospective ECG-gating was taken to reduce the movement artifact. The region of interests (ROIs) for monitoring the contrast was placed in the aortic arch. The threshold values were 150 Hounsfield unit (HU). After the CT values arrived at the threshold values and delay 5 seconds, the scans automatically started. The main parameters are as follows: tube voltages 100-120 kV (adapted to body mass index, tube current 220mAs, gantry rotation time 0.25s, pitch 0.2-0.5(according to heart beat), slice thickness 0.625mm, convolution kernel B26f.

| **Supplementary Table 1**. **Radiomics features extracted from cardiovascular CECT** | | |
| --- | --- | --- |
| **Group** | **No.** | **Radiomics features** |
| GLCM | N1-N330 | GLCM describes general information of intensity levels of CT images in different directions and offsets and arrangement pattern of different voxels. In this study, each GLCM feature was measured by four directions (*θ* = 0°, 45°, 90°, 135°) and three offsets (*d* = 1, 4, 7), including the following: autocorrelation, difference entropy, cluster prominence (CP), cluster shade (CS), contrast, correlation, difference entropy (DE),dissimilarity, energy, entropy, homogeneity 1, homogeneity 2, inverse diff moment norm (IDMN), inverse diff norm (IDN), inverse variance (IV), Information Measure Correlation 2 (IMC2), max probability (MA), sum average (SA), sum entropy (SE), sum variance (SV) and variance. |
| GLRLM | N331-N363 | GLRLM quantifies gray level runs in CT images, which is mainly used for analysis of linear structure. In this study, each GLRLM feature was measured by two directions (θ = 0°, 90°), including the following: gray level nonuniformity (GLN), high gray level run emphasis (HGLRE), long run emphasis (LRE), long run high gray level emphasis (LRHGE), long run low gray level emphasis (LRLGLE), low gray level run emphasis (LGLRE), run length nonuniformity (RLN), run percentage (RP), short run emphasis (SRE), short run high gray level emphasis (SRHGLE) and short run low gray level emphasis (SRLGLE). |
| Intensity histogram | N364-N412 | Intensity histogram describes distributing characteristics of voxel intensities from CT images based on commonly used and basic metrics, including the following: interquartile range (IR), kurtosis, mean absolute deviation (MAD_1_), median absolute deviation (MAD_2_), percentile, percentile area (PA), quantile, range and skewness. |
| Shape | N413-N430 | Shape presents descriptors of the three-dimensional size and shape of the tumor region, including the following: compactness1, compactness2, convex, convex hull volume (CHV), convex hull volume 3D (CHV3D), mass, max 3D diameter (M3DD), mean breadth (MB), number of objections (NOB), number of voxels (NOV), orientation, roundness, spherical disproportion (SD), sphericity, surface area (SA), surface area density (SAD), voxel size ( VS) and volume. |
| Abbreviations: IR, intensity histogram; GLCM, gray level co-occurrence matrix; GLRLM, gray level run length matrix. | | |

| **Supplementary Table2. Information of four pairs of radiomics features with strong positive correlation** | | | | |
| --- | --- | --- | --- | --- |
| **No.** | **Features** | **parameters** | **Coefficients** | **p** |
| 102 | Homogeneity2 | *θ* = 45, d=7 | 0.988 | <0.001 |
| 119 | IV | *θ* = 45, d=7 |  |  |
| 102 | Homogeneity2 | *θ* = 45, d=7 | 0.963 |  |
| 245 | MP | *θ* = 135, d=7 |  |  |
| 186 | IMC2 | *θ* = 333, d=1 | 0.949 |  |
| 189 | IMC2 | *θ* =90, d=1 |  |  |
| 119 | IV | *θ* = 45, d=7 | 0.938 |  |
| 245 | MP | *θ* = 135, d=7 |  |  |
| Abbreviations: IV, Inverse Variance; IMC2, Information Measure Corr2; MP, Max Probability; | | | | |

| **Supplementary Table 3. Information of eight selected radiomics features** | | |
| --- | --- | --- |
| **No.** | **features** | **parameters** |
| 28 | CP | *θ* = 45, d=7 |
| 118  158 | IV  Roundness | *θ* = 45, d=7 |
| 189 | IMC2 | *θ* =90, d=1 |
| 284 | MAD |  |
| 286 | 10Percentile |  |
| 314 | Compactness1 |  |
| 320 | SAD |  |
| Abbreviations: CP, Cluster Prominence; IR, Inverse Variance; IMC2: Information Measure Corr2; MAD, Median Absolute Deviation; SAD, surface area density. | | |
